# Silent Harm: How Ambient Air Pollution Threatens Prenatal and Neonatal Health. A Systematic Review

**DOI:** 10.17533/udea.iee.v43n3e08

**Published:** 2025-10-25

**Authors:** Dr. Ranjana Chavan, Dr. Jasneet Kaur

**Affiliations:** 1 Assistant Professor, Ph.D. Email: ranjanachavan@scon.edu.in. https://orcid.org/0000-0001-6246-7886 Symbiosis International University India ranjanachavan@scon.edu.in; 2 Professor, Ph.D. Email: jasneetkaur@scon.edu.in. https://orcid.org/0000-0001-6897-9137 Symbiosis International University India jasneetkaur@scon.edu.in; 3 Symbiosis College of Nursing, Symbiosis International (Deemed University), Pune, India. Symbiosis International University Symbiosis College of Nursing Symbiosis International (Deemed University) Pune India

**Keywords:** ambient air pollution, neonatal outcomes, pregnancy complications, particulate matter, maternal health., contaminación del aire ambiente, resultados neonatales, complicaciones del embarazo, material particulado, salud materna., poluição do ar ambiente, desfechos neonatais, complicações na gravidez, material particulado, saúde materna.

## Abstract

**Objective.:**

To examine the link between ambient air pollution and poor pregnancy and neonatal outcomes.

**Methods.:**

This systematic study searched numerous databases, including PubMed, Scopus, Web of Science, and Cochrane Library, revealed 26 papers that met established criteria. This research looked at how pollutants such as Particulate matter smaller than 2.5 microns, Particulate Matter ≤10 micrometers, Nitrogen Dioxide, Sulfur Dioxide, Ozone, and black carbon affected maternal and new-born health, including miscarriage, preeclampsia, preterm delivery, low birth weight, and neonatal respiratory and neurological abnormalities.

**Results.:**

Findings repeatedly revealed that enhanced the danger of gestational problems & poor neonatal consequences, with pollutants including Particulate matter smaller than 2.5 microns and Nitrogen Dioxide substantially related to hypertensive disorders, before the expected time of delivery, low birth weight, and reduced new-born immune and respiratory function. The paper also discusses how pollution impacts health via biological processes such as oxidative stress and epigenetic alterations. Variability in research designs, exposure assessment methodologies, and regional pollution levels were observed.

**Conclusion.:**

This review underscores the link between ambient air pollution, particularly Particulate matter smaller than 2.5 microns and Nitrogen Dioxide, and poor pregnancy and neonatal outcomes. Recognizing these risks is crucial for nursing care, allowing nurses to educate, identify early risks, and advocate for policies that protect mothers and newborns. Strengthening interventions will improve health outcomes for both.

## Resumen

**Objetivo.:**

Examinar la relación entre la contaminación del aire en ambiente y los resultados adversos prenatales y neonatales.

**Métodos.:**

Revisión sistemática en la que se buscó información en bases de datos, entre ellas PubMed, Scopus, Web of Science y Cochrane Library. Esta investigación analizó cómo los contaminantes, como las partículas de menos de 2.5 micras, las partículas de ≤10 micras, el dióxido de nitrógeno, el dióxido de azufre, el ozono y el carbono negro, afectaban a la salud materna y neonatal, incluyendo abortos espontáneos, preeclampsia, partos prematuros, bajo peso al nacer y anomalías respiratorias y neurológicas neonatales**.**

**Resultados.:**

Se analizaron 26 artículos que cumplían los criterios establecidos. Los hallazgos revelaron repetidamente que la contaminación ambiental aumentaba el riesgo de problemas gestacionales y de consecuencias neonatales adversas; y que contaminantes como las partículas de menos de 2.5 micras y el dióxido de nitrógeno estaban relacionados de manera significativa con trastornos hipertensivos, partos antes de la fecha prevista, bajo peso al nacer y disminución de la función inmunológica y respiratoria del recién nacido. También se analizó cómo la contaminación afecta la salud a través de procesos biológicos como el estrés oxidativo y las alteraciones epigenéticas. Se observó variabilidad en los diseños de las investigaciones, las metodologías de evaluación de la exposición y los niveles de contaminación regional.

**Conclusión.:**

Esta revisión subraya la relación entre la contaminación del aire, en particular las partículas de menos de 2.5 micras y el dióxido de nitrógeno, con los resultados adversos prenatales y neonatales. Reconocer estos riesgos es fundamental para la atención de enfermería, ya que permite a las enfermeras educar, identificar los riesgos tempranos y promover políticas que protejan a las madres y a los recién nacidos. El fortalecimiento de las intervenciones mejorará los resultados de salud para ambos.

## Resumo

**Objetivo.:**

Examinar a relação entre poluição do ar ambiente e desfechos pré-natais e neonatais adversos.

**Métodos.:**

Foi realizada uma revisão sistemática com busca de informações em bases de dados como PubMed, Scopus, Web of Science e Biblioteca Cochrane. Este estudo analisou como poluentes, como material particulado menor que 2.5 mícrons, material particulado ≤ 10 mícrons, dióxido de nitrogênio, dióxido de enxofre, ozônio e carbono negro, afetaram a saúde materna e neonatal, incluindo abortos espontâneos, pré-eclâmpsia, parto prematuro, baixo peso ao nascer e anormalidades respiratórias e neurológicas neonatais**.**

**Resultados:**

. Vinte e seis artigos que atenderam aos critérios estabelecidos foram analisados. Os resultados revelaram repetidamente que a poluição do ar ambiente aumentou o risco de problemas gestacionais e desfechos neonatais adversos; e que poluentes como material particulado menor que 2.5 mícrons e dióxido de nitrogênio foram significativamente associados a distúrbios hipertensivos, parto prematuro, baixo peso ao nascer e diminuição da função imunológica e respiratória do recém-nascido. Também analisamos como a poluição afeta a saúde por meio de processos biológicos, como estresse oxidativo e alterações epigenéticas. Variabilidade foi observada em delineamentos de pesquisa, metodologias de avaliação de exposição e níveis regionais de poluição.

**Conclusão.:**

Esta revisão destaca a relação entre poluição do ar, particularmente material particulado menor que 2.5 mícrons e dióxido de nitrogênio, com desfechos pré-natais e neonatais adversos. Reconhecer esses riscos é fundamental para o cuidado de enfermagem, pois permite que os enfermeiros eduquem, identifiquem riscos precoces e promovam políticas que protejam mães e recém-nascidos. O fortalecimento das intervenções melhorará os desfechos de saúde para ambos.

## Introduction

Ambient air pollution, a major global health issue, is one of the leading causes of premature death and disease burden. It causes 6.7 million deaths globally, making it the third most significant risk factor for death.^1^ The World Health Organisation (WHO) describes pollution in the air as any chemical, physical, or biological agent that contaminates the interior or outdoor environment and alters the atmosphere's inherent properties.^2^ Rapid urbanization, industry, and high vehicle emissions are major causes of air pollution. The elevated amounts of greenhouse pollutants, The combustion of fossil fuels has resulted in a rise in carbon dioxide, methane, nitrous oxide, ozone , and fluorinated gases.^3^ These heat-trapping GHGs increase the frequency and severity of wildfires, sand and dust storms, and global surface temperatures, as well as contributing to air pollution, particularly particulate matter (PM), a complex mixture of solids and aerosols varying in size, shape, and chemical composition. PM is made up of metals, dust or soil particles, natural and synthetic compounds, and allergens.^4^ Those that have an aerodynamic diameter of less than 10µm (Particulate Matter ≤10 micrometers), which is small enough to penetrate the lungs and be deposited in the upper airways, are especially concerning. Aerodynamic diameters smaller than 2.5µm can enter the circulatory system via the alveoli of the lungs (Particulate matter smaller than 2.5 microns) are considerably more dangerous.^5^

The World Health Organization (WHO) suggests that air pollution causes around seven million premature mortality per year primarily due to respiratory and cardiovascular diseases. Furthermore, 99 percent of people worldwide breathe air that exceeds permissible limits, with low- and middle-income countries bearing the brunt of this burden.^6^ Ambient air pollution has a more harmful impact on vulnerable individuals, including children, the elderly, pregnant women, and people with comorbidities. ^1^^,^^7^^,^^8^ Because of their special physiology, expectant mothers and their unborn kids are especially vulnerable to the impacts of air pollution. Physiological changes during pregnancy include a 20% increase in oxygen intake, a 40% to 50% increase in minute breathing, and a 40% increase in cardiac output.^9^ These modifications raise exposure by increasing the quantity of pollutants that are breathed in and circulated. The new-born is especially at risk and may have already been negatively affected by air pollution while still in the womb. Pollutants cross the placenta and reach the fetal circulation. According to a study, carbonaceous air pollution particles breathed by mothers during pregnancy had the capability to enter through the placenta and enter embryonic organs.^10^ Once in the maternal circulation, these pollutants activate many biological processes that affect prenatal and neonatal health. Oxidative stress is a primary mechanism in which pollution exposure produces reactive oxygen species (ROS) that damage placental and fetal cells, increasing the likelihood of problems such preeclampsia, gestational hypertension and intrauterine growth restriction.^11^

Pollutant exposures such as Particulate matter smaller than 2.5 microns and Nitrogen Dioxide triggers pro-inflammatory cytokines such as IL-1β and TNF-α, which can damage placental function and raise the risk of premature delivery.^12^^,^^13^ Pollutant exposure also causes placental malfunction, since alterations in vascularization and nutritional transport pathways can result in fetal hypoxia and malnutrition, raising the risk of low birth weight , preterm birth^14^ and young kids death, and adverse lung and respiratory effects.^15^ Air pollution has also been linked to epigenetic alterations in placental DNA, such as changes in DNA methylation patterns that affect gene expression during fetal development,^16^ These changes might have long-term effects on the health of the children.^17^ Notably, individuals trying to conceive are also at significant risk for prolonged time to conception,^18^ miscarriage,^19^^-^^21^ infertility^22^ and decreased success rate with in-vitro fertilization (IVF) treatment due to the effects of high air pollution exposure.^23^

In addition to gestational exposures, new-borns’ lungs are still developing, making them susceptible to airborne pollutants.(24) Neonatals have a greater resting metabolic rate than older children and adults, resulting in increased oxygen consumption, upper and lower airway resistance, reduced lung capacity, and respiratory muscle endurance.^25^ Because infants breathe in twice as much air per body weight as adults do, twice as many air contaminants may potentially enter an infant's lungs. Since newborns' airways are smaller, even a slight restriction brought on by inflammation from exposure to environmental contaminants or infection in lung has a unreasonable effect on airway obstruction. ^26^^) (^^27^

Despite the well-established harmful health effects of air pollution of expectant mothers and new-borns, especially in low-income areas where data collection and air quality monitoring are few, there are still research gaps. Furthermore, although a number of studies have examined links between air pollution and unfavourable birth outcomes, more longitudinal research is required to demonstrate direct pathways, and causative mechanisms are currently being examined^.(^^28^ With an emphasis on developing nations, where the burden of pollution-related health issues is disproportionately large, this study attempts to methodically evaluate the available data regarding the effects of ambient air pollution on maternal and neonatal outcomes. 

The objective of this review was to assess the relationship among ambient air pollution and adverse prenatal and neonatal outcomes. It examines the impact of key pollutants (Particulate matter smaller than 2.5 microns, Particulate Matter ≤10 micrometers, Nitrogen Dioxide, Sulfur Dioxide, Ozone) on maternal and neonatal health, explores underlying biological mechanisms, and identifies key research gaps and future directions for intervention.

## Methods

The aim of this systematic review is to put together what is now known about how air pollution affects pregnancy and neonatal outcomes. The study looks at a range of health outcomes for pregnant women and new-borns, including preeclampsia, pregnancy-related hypertension, premature birth, low birth weight, intrauterine growth restriction, and new-born respiratory distress syndrome. To enhance methodological transparency and reproducibility, this systematic review follows the Preferred Reporting Items for Systematic Reviews and Meta-Analyses (PRISMA) recommendations.^29^ It provides a formal framework for performing and reporting systematic reviews, guaranteeing that the review process is complete, neutral, and methodologically sound.

**Figure 1:**
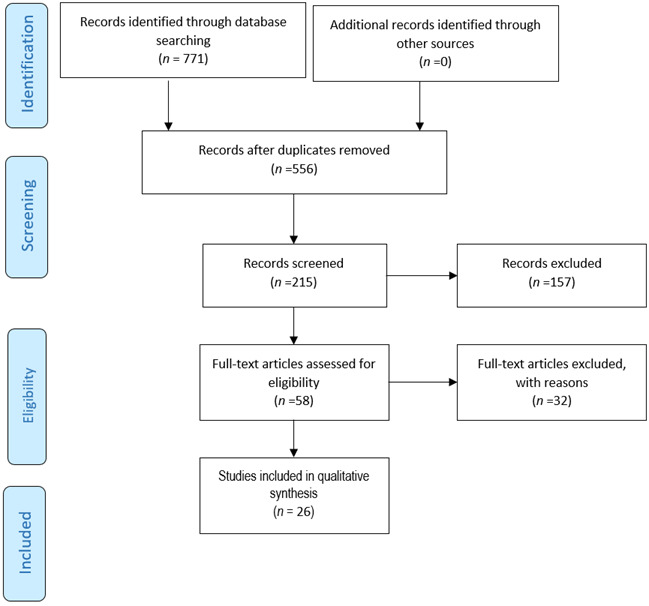
Schematic presentation of PRISMA flowchart

Eligibility criteria. *(i) Inclusion criteria*. The individuals selected for this review must meet the PECOS criteria: Pregnant women during all stages of their pregnancy, including birth; Prenatal exposure to air pollution is one example of an exposure; Pregnant women had less exposure levels, with or without bad delivery results, compared to those with higher exposure and unfavorable birth outcomes; Unfavourable birth outcomes include birth of neonate before expected date, low birth weight, and perinatal death; Furthermore, papers written in English, research conducted between 2018 and 2024 were included. *(ii) Exclusion criteria.* Research Article lacking a full report; Qualitative studies, systematic reviews, short communications, and commentaries were excluded; and Studies focusing on occupational or indoor air pollution.

Search strategy. To study the relationship of environmental air pollution & maternal & neonatal consequences, a comprehensive search strategy was developed. The primary research question focused on how exposure associate ambient air pollution with gestation and outcomes of neonates such as birth weight, preterm birth, and neonatal mortality. Searches were conducted in major scientific databases, including PubMed, Scopus, Web of Science, Cochrane Library, and ScienceDirect. Search queries combined these terms using for example: ((“Air pollution, ”[MeSH Terms] OR(“Ambient Particulate Matter”[MeSH Terms] OR(“Traffic-Related Pollution”[MeSH Terms]))) AND ”[Title/Abstract] ((“Maternal Health”*OR “Pregnancy Outcome”* (Pre-Eclampsia, Eclampsia, Diabetes, Gestational, Abortion, Spontaneous]) [Title/Abstract] “AND (((“Newborn Health”* OR Infant Health* ((“Infant, Low Birth Weight”[Title/Abstract] OR “Premature Birth ” [Title/ Abstract] OR “Cerebral Palsy”[Title/Abstract]))). PRISMA flowchart was employed to document study selection

Study Selection. In order to find pertinent papers on the outcome of environment air pollution on pregnant & newborn outcomes, two researchers independently carried out a thorough search. The chosen studies were also evaluated for methodological rigor and relevance. Full-text publications of the pertinent research were obtained and carefully examined in order to guarantee the review's objectivity and legitimacy. Each study's eligibility was assessed independently by two researchers, lowering the possibility of selection bias. To ensure openness and consistency in the selection process, the procedure involved carefully recording the justifications for rejecting research that did not fit the predetermined standards. This thorough method improves the validity and dependability of the review's conclusions while also being in line with standards for research quality.

Data extraction. An organized data extraction form was created in order to methodically gather important data from chosen studies. The retrieved data contained research study features such as author, year of publication, country, research design, and sample size. Exposure measures were also recorded, detailing the type of pollutant, measurement methods and exposure duration. Additionally, relevant outcome factors such as gestational age at birth, birth weight, neonatal death, congenital anomalies, stillbirth, intrauterine growth restriction, neonatal respiratory distress were systematically documented. To ensure consistency and minimize bias, two reviewers independently extracted the data. Any disagreements arising during the process were resolved through discussion.

Quality of Bias. The Joana Briggs Institute (JBI) critical assessment checklist for cohort and case-control studies (30) was utilized to evaluate the standard of the appropriate studies. Two reviewers did an independent quality evaluation. Any inconsistencies discovered during the quality evaluation were resolved through evidence-based conversations with the assistance of a third researcher. Only papers with a quality evaluation score of more than 50% were chosen for this review, ^30^^) (^^31^ as shown in Table 1. All studies got score from 7 to 10. One cross-sectional study ^32^ included in this review had a poorer quality score (4/11) based on the Joanna Briggs Institute criteria, owing mostly to its research design. Regardless of its shortcomings, this study provides important early information on the association of air pollution in the environment and outcomes of neonatal. Interestingly, its findings are similar with those of higher-quality cohort studies included in this review, indicating an overall trend of increased risk linked with particulate matter (Particulate matter smaller than 2.5 microns, Particulate Matter ≤10 micrometers) & nitrogen dioxide (Nitrogen Dioxide). 

Search Results. A total of 771 studies from PubMed, Scopus, Web of Science, Cochrane Library, Science Direct, were found through the methodical search. There were 215 papers left for title and abstract screening after duplicates 556 studies were eliminated. Studies that were deemed irrelevant to ambient air pollution and maternal or neonatal outcomes were eliminated after the first screening procedure 157. Predetermined inclusion and exclusion criteria were used to assess eligibility of the complete texts of 58 research. Following the full-text evaluation, papers with methodological flaws, insufficient data, or no pertinent exposure assessment were disqualified 32. Lastly, the studies included 26 studies as presented in the PRISMA flowchart (Figure 1). 

**Table 1:** Features of the research articles

Sr. No	Cite	Author /Year /Country	Title	Research design	Samples	Exposure	Research findings
	(33)	Gaskins *et al.*, 2019 USA	Pollution in the Air and Miscarriage Threat	Prospective cohort study	19308	Proximity to roads and PM	Air pollution linked to increased miscarriage risk in sensitive subgroups.
	(34)	Mitku *et al*., 2023 South Africa	Impact of Ambient Air Pollution during Pregnancy on Birth Outcomes	Mother and Child in the Environment birth cohort	996	Particulate matter smaller than 2.5 microns, Sulfur Dioxide	Preterm birth, low birthweight/ Small for gestational age
	(35)	Sun *et al*., 2024 USA	Exposure during pregnancy of Black Carbon Particles and Fetal Impact	Retrospective cohort study	386 361	Particulate matter smaller than 2.5 microns, black carbon, and organic matter	Increased Preeclampsia -Eclampsia risk associated with exposure.
	(36)	Yuan *et al*., 2023 China	Pollution of air and Hypertension during the Pregnancy	Cohort Study	22 821	Particulate matter smaller than 2.5 microns and PM1	Increased de novo hypertensive disorders of pregnancy risk, especially in early pregnancy.
	(37)	Niedzwiecki *et al*., 2020 Mexico	Air Pollution and Postpartum Depression	Cohort study	509	Prenatal and postpartum exposure to Particulate matter smaller than 2.5 microns	Higher postpartum depression risk at six months
	(38)	Duan *et al.,* 2022 China	Air Pollution and Postpartum Depression Risk	Cohort study	10 209	Particulate matter smaller than 2.5 microns, Particulate Matter ≤10 micrometers, sulfer oxide Carbon Monoxide, Nitrogen Dioxide, and Ozone	Increased exposure during pregnancy significantly elevated postpartum depression risk at 6 months
	(39)	Bastain *et al*., 2021 US	Prenatal Air Pollution and Maternal Depression	Maternal and developmental risks from environmental and social stressors cohort	800	Nitrogen Dioxide, Particulate matter smaller than 2.5 microns, and Particulate Matter ≤10 micrometers	Increased second trimester exposure linked to postpartum depression.
	(40)	Ananth *et al.*, 2018 USA	Air Pollution and Placental Abruption Risk	Case-crossover design	1190	Nitrogen Dioxide, Particulate matter smaller than 2.5 microns	Specific lag days increased the odds of abruption.
	(41)	Cocchi *et al*., 2023 taly	Air Pollution and Aeroallergens as Preterm Birth Triggers	Retrospective cohort design	Not specified	Particulate matter smaller than 2.5 microns**,** Ozone**,** Nitrogen Dioxide**,** and aeroallergens	Acute air pollution exposure prior to delivery linked to increased preterm birth risk.
	(42)	Yu *et al*., 2020 China	Maternal Particulate matter smaller than 2.5 microns Exposure and GDM"	Cross sectional study	54 517	Particulate matter smaller than 2.5 microns	Increased exposure during the 2nd trimester was linked to higher Gestational Diabetes Mellitus risk.
	(43)	Gaskins *et al*., 2020 England	Air Pollution and Pregnancy Loss Risk in assisted reproductive technologies	Prospective cohort study	275	Particulate matter smaller than 2.5 microns, Black Carbon, Nitrogen Dioxide, Ozone	Higher exposure of Nitrogen Dioxide after 30 days of pregnancy was linked to increased loss risk
	(44)	Xu *et al*., 2023 China	Air Pollution, Pregnancy Hormones, and Early Miscarriage	Case-control study	440	Carbon Monoxide, Sulfur Dioxide	Short-term exposure increases early miscarriage risk via progesterone changes.
Neonatal Outcomes							
	(45)	He *et al.*, 2019 China	Early-Life Air Pollution and Lung Function	Cohort Study	2942	Particulate Matter ≤10 micrometers, Nitrogen Dioxide, Nitrogen oxide	Reduced lung function and increased wheezing risk.
	(46)	Pedersen *et al*., 2023 Denmark	Prenatal Air Pollution and Immune Disruptions	Prospective mother-child cohort	700	Nitrogen Dioxide, Particulate matter smaller than 2.5 microns, Particulate Matter ≤10 micrometers	Prenatal exposure associated with immune changes, allergies, and asthma risk at 6 years of age of Child
	(47)	Martins Costa Gomes *et al*., 2021 US	Antenatal Air Pollution Linked to Altered Cord Blood Immunity	Prospective cohort design	91	Particulate matter smaller than 2.5 microns, Sulfur Dioxide	Gestational vulnerability associated with immune cell alterations in newborns.
	(48)	García-Serna *et al*., 2021 Spain	Traffic Pollution in pregnancy Impairs Newborn Immunity	New-borns and Environmental Air Pollution Cohort	NA	Nitrogen Dioxide, Particulate matter smaller than 2.5 microns, Particulate Matter ≤10 micrometersOzone	Prenatal Air Pollution and Immune Cell Changes
	(49)	Madhloum *et al*., 2019 Belgium	Prenatal Air Pollution and Blood Pressure of Neonates	Prospective Birth Cohort Study	427	Particulate matter smaller than 2.5 microns, Particulate Matter ≤10 micrometers Black Carbon, Nitrogen Dioxide	Prenatal air pollution exposure linked to higher newborn BP
	(50)	Ghazi *et al*., 2021 South Africa	Air Pollution, Placental Methylation, and Foetus Health	Epidemiological cohort study	Not specified	Particulate matter smaller than 2.5 microns, Particulate Matter ≤10 micrometers Nitrogen Dioxide, Ozone, Black carbon	Prenatal air pollution alters placental epigenetics, impacting fetal health
	(51)	Gu *et al.*, 2024 China	Association between environmental pollutants and the risk of premature infant	Retrospective cohort study	7288	Particulate matter smaller than 2.5 microns, Particulate Matter ≤10 micrometers, Nitrogen Dioxide, Sulfur Dioxide, Carbon Monoxide	Exposure to air pollution during pregnancy is strongly linked to an increased risk of preterm birth
	(32)	Kaiser *et a*l., 2023 Austria	Prenatal exposure to per- and polyfluoroalkyl substances Exposure and Pregnancy Outcomes	Cross-sectional study	136	Prenatal exposure to per- and polyfluoroalkyl substances	Prenatal exposure to per- and polyfluoroalkyl substances exposure was linked to placental Perfluorodecanoic acid in Small for gestational age births and Perfluorohexane sulfonic acid in preterm birth
	(52)	McGuinn *et al.*, 2020 Mexico	Prenatal Particulate matter smaller than 2.5 microns Exposure and Child Behavior	Prospective birth cohort	539	Particulate matter smaller than 2.5 microns	Prenatal first-trimester Particulate matter smaller than 2.5 microns exposure was linked to higher attention problem and hyperactivity scores.
	(53)	Shih *et al.*, 2020 Taiwan	Prenatal Traffic Pollution and Hyperactivity"	Birth Cohort Study	16 376 mother-infant pairs	Nitrous Oxide	Prenatal nitric oxide exposure significantly increased hyperactivity risk in children before age eight.
	(54)	Irizar *et al*., 2021 Spain	Prenatal Air Pollution and Newborn Thyroxine	Prospective cohort study	463	Particulate matter smaller than 2.5 microns, Nitrogen Dioxide	Prenatal Particulate matter smaller than 2.5 microns exposure increased newborn TT4 levels, with stronger effects later in pregnancy
	(15)	Johnson *et al.*, 2024 Canada	Prenatal Pollution and Newborn Respiratory Distress	Prospective cohort study	2001	Particulate matter smaller than 2.5 microns, Nitrogen Dioxide	Pollution exposure increases neonatal interventions, particularly ventilation and antibiotic treatments
	(55)	Y. Zhang *et al*., 2024 Canada	Prenatal Pollution and Cerebral Palsy Risk	Cohort study	1 587 935	Particulate matter smaller than 2.5 microns, Nitrogen Dioxide, Ozone	Higher prenatal Particulate matter smaller than 2.5 microns linked to increased cerebral palsy risk in newborns.
	(56)	Soesanti *et al.*, 2023 Indonesia	Traffic Pollution Effects on Birth Size	Prospective cohort study	413	Particulate matter smaller than 2.5 microns, soot, Nitrogen Oxide, and Nitrogen Dioxide	High air pollution linked to shorter birth length but not weight.

**Abbreviations:** PM 2.5 microns: Particulate matter smaller than 2.5 microns; SO2 Sulfur Dioxide; PM10 - Particulate Matter ≤10 micrometers; NO: Nitric Oxide; CO - Carbon Monoxide; NO2 - Nitrogen Dioxide; O3 - Ozone; MACE: Mother and Child in the Environment; HDP: HF; Black Carbon (BC); MADRES Maternal and developmental risks from environmental and social stressors; NELA: New-borns and Environmental Air Pollution; ENVIRONAGE (ENVIRonmental influence ON early AGEing); ART: assisted reproductive technologies; PFAS: prenatal exposure to per- and polyfluoroalkyl substances

## Results

Around 26 research articles were finalized as per criteria developed by researcher. The results of these studies are more broadly applicable because they were carried out in a variety of geographical locations, such as the USA (5), China (5), South Africa (2), Mexico (2), Spain (2), Belgium (1), Canada (2), Denmark (1), Taiwan (1), Indonesia (1), Italy (1), Austria (1), and England (1). With 528802 participants, the entire sample size offers strong evidence for the link between prenatal exposure to atmospheric air pollution and unfavourable gestational and new-born consequences. According study's findings, exposure to pollutants like Particulate matter smaller than 2.5 microns, Particulate Matter ≤10 micrometers, Nitrogen Dioxide, Sulfur Dioxide, Ozone, Carbon Monoxide, and black carbon poses serious risks for miscarriage, hypertensive disorders of pregnancy, gestational diabetes mellitus, postpartum depression, preterm birth, low birth weight, nervous system dysfunction, immunological dysfunction, and neurodevelopmental disorders in new-borns. These findings underscore the urgent need for targeted policies and interventions to mitigate the harmful outcomes of air pollution on mother and new-born health.

### Maternal Outcomes

Miscarriage & Pregnancy Loss. Gaskins *et al.*^21^ performed a prospective cohort study in the USA include 19 308 expectant mothers. Their research discovered that exposure to air pollution, particularly fine particulate matter (Particulate matter smaller than 2.5 microns) and proximity to roads, significantly augmented the danger of miscarriage. This effect was more pronounced in sensitive subgroups, such as those with previous health problems. Likewise, Xu *et al*^44^ China performed a case-control study with 440 participants and reported that early contact to carbon monoxide and sulfur dioxide (Sulfur Dioxide) linked with early abortion. They suggested that air pollution might disrupt hormone levels, particularly progesterone, leading to pregnancy loss. Furthermore, Gaskins *et al.*^43^ in England studied 275 ladies experienced assisted reproductive technologies. They found that greater prominence to nitrogen dioxide (Nitrogen Dioxide) and Particulate matter smaller than 2.5 microns after 30 days of pregnancy significantly increased the risk of pregnancy loss, highlighting the vulnerability of assisted reproductive technologies pregnancies to air pollution.

Pregnancy Hypertension & Preeclampsia. Yuan *et al.*^36^ in China conducted a cohort study involving 22 821 women and found that vulnerability to Particulate matter smaller than 2.5 microns and PM1 during early pregnancy was connected to a more risk of de novo hypertensive disorders of pregnancy. Their study emphasized that the risk was modified by age of the mother and education status, suggesting socioeconomic factors might influence susceptibility. Similarly, Sun *et al.*^35^ in the USA analysed data from 386 361 pregnancies in a retrospective cohort study and found vulnerability to black carbon, Particulate matter smaller than 2.5 microns, and organic matter highly danger of preeclampsia and eclampsia. This study exhibits the part of air pollution in maternal cardiovascular complications, which can lead to life-threatening conditions if untreated.

Placental & Birth Complications. Ananth *et al.*^40^ in the USA conducted a case-crossover study on 1190 women and found that vulnerability to Nitrogen Dioxide & Particulate matter smaller than 2.5 microns on specific lag days significantly increased the odds of placental abruption. Placental abruption is a severe pregnancy complication leading to heavy bleeding and potential foetal distress. Kaiser *et al*.^32^ in Austria investigated a cross-sectional study on 136 pregnancies and found that prenatal exposure to per- and polyfluoroalkyl substances was linked to small-for-gestational-age births and preterm birth. Ghazi *et al.*^50^ in South Africa explored the molecular mechanisms behind these outcomes and discovered that prenatal vulnerability to Particulate matter smaller than 2.5 microns, Particulate Matter ≤10 micrometers, Nitrogen Dioxide, and Ozone led to DNA methylation changes in the placenta. These epigenetic alterations can impact fetal development, birth outcomes, and long-term disease susceptibility

Postpartum Depression. Niedzwiecki *et al*.^37^ in Mexico conducted a cohort study with five zero nine women discovered the prenatal and postpartum vulnerability to Particulate matter smaller than 2.5 microns significantly highly danger of postpartum depression at six months. A similar study by Duan *et al*.^38^ in China involving 10 209 women confirmed these findings, reporting that vulnerability to Particulate matter smaller than 2.5 microns, Particulate Matter ≤10 micrometers, Sulfur Dioxide, Nitrogen Dioxide, and Ozone during gestation heightened postpartum depression risk. Bastain *et al*.^39^ in the USA studied 800 women in the Maternal and developmental risks from environmental and social stressors cohort and discovered that increased vulnerability to Nitrogen Dioxide, Particulate matter smaller than 2.5 microns, & Particulate Matter ≤10 micrometers throughout the second trimester particularly related to postpartum depression, suggesting that mid-pregnancy is a critical period of susceptibility.

Gestational Diabetes. Yu *et al.*^42^ in China conducted a cross-sectional study with 54 517 women and discovered that increased vulnerability to Particulate matter smaller than 2.5 microns throughout the 2^nd^ trimester was significantly more prone of gestational diabetes mellitus (GDM). This finding suggests that air pollution may provide to metabolic disorders in pregnancy, potentially affecting both maternal and fetal health.

### Neonatal Outcomes

Premature Birth & Underweight. Mitku *et al*.^34^ in South Africa conducted a birth cohort study with 996 participants and discovered vulnerability to Particulate matter smaller than 2.5 microns and Sulfur Dioxide was related with more frequencies of preterm birth and underweight, particularly small-for-gestational-age infants. Cocchi *et al.*^41^ in Italy performed a retrospective cohort study and discovered that acute vulnerability to Particulate matter smaller than 2.5 microns, Ozone, Nitrogen Dioxide, and aeroallergens just before delivery significantly increased the risk of preterm birth. Similarly, Gu *et al* ^.(^^51^ in China analysed 7288 pregnancies and found that exposure to Particulate matter smaller than 2.5 microns, Particulate Matter ≤10 micrometers, Nitrogen Dioxide, Sulfur Dioxide, and Carbon Monoxide throughout gestation substantially increased the risk of preterm birth. Soesanti *et al*.^56^ in Indonesia studied 413 new-borns and discovered that high vulnerability to Particulate matter smaller than 2.5 microns, Nitric Oxide, and Nitrogen Dioxide related with shorter birth duration, although birth weight was not significantly affected.

Immune System & Allergy Risks. Pedersen *et al.*^46^ in Denmark conducted a prospective mother-child cohort study with 700 participants and established that antenatal vulnerability to Nitrogen Dioxide, Particulate matter smaller than 2.5 microns, and Particulate Matter ≤10 micrometers was linked to immune system disruptions, leading to increased risks of allergies and asthma in kids at the 6 years of age. Martins Costa Gomes *et al.*^47^ in the USA studied 91 new-borns and found that prenatal exposure to Particulate matter smaller than 2.5 microns and Sulfur Dioxide altered immune cell profiles in cord blood, potentially affecting the child’s long-term immunity. Similarly, García-Serna *et al*. ^48^ in Spain analysed data from the New-borns and Environmental Air Pollution cohort and found that traffic-related air pollution, particularly Nitrogen Dioxide, Particulate matter smaller than 2.5 microns, Particulate Matter ≤10 micrometers, and Ozone, impaired new-born immune function by altering immune cell counts

Respiratory and Neurological Risks. He *et al.*^45^ in China conducted a cohort study with 2942 infants and found that prenatal and early postnatal vulnerability to Particulate Matter ≤10 micrometers and Nitrogen Dioxide reduced respiratory activities and increased wheezing risk. Johnson *et al.*^15^ in Canada studied 2,001 new-borns and discovered that vulnerability to Particulate matter smaller than 2.5 microns and Nitrogen Dioxide significantly enhanced the likelihood of neonatal respiratory distress, often demanding management such as ventilation and antibiotic treatments. Y. Zhang *et al*^55^ in Canada performed a large cohort study with 1 587 935 births and identified that antenatal ventilation to Particulate matter smaller than 2.5 microns was related to an increased risk of cerebral palsy, highlighting the prone to get neurotoxic impacts of air pollution. 

Neurodevelopmental Effects. McGuinn *et al.*^52^ in Mexico performed a prospective birth cohort study with 539 children & discovered that first-trimester exposure to Particulate matter smaller than 2.5 microns was related with higher attention problem & hyperactivity scores. Shih *et al.*^53^ in Taiwan studied 16 376 mother-infant pairs and found that prenatal exposure to nitric oxide suggestively amplified the danger of hyperactivity in kids before the age of eight. Madhloum *et al.*^49^ in Belgium conducted a prospective birth cohort study with 427 new-borns and vulnerability during gestation to Particulate matter smaller than 2.5 microns, Particulate Matter ≤10 micrometers, black carbon & Nitrogen Dioxide was linked to more neonatal blood pressure. Irizar *et al.*^54^ in Spain studied 463 pregnancies and found that prenatal Particulate matter smaller than 2.5 microns exposure increased newborn thyroxine (TT4) levels, with stronger effects when exposure occurred later in pregnancy, suggesting a possible disruption in thyroid function

## Discussion

A full look at the data shows that pollution in the air around us has a big effect on the health of mothers and their babies. The results show that being around pollutants like Particulate matter smaller than 2.5 microns, Particulate Matter ≤10 micrometers, Nitrogen Dioxide, Sulfur Dioxide, and black carbon is strongly linked to bad pregnancy outcomes like miscarriage, high blood pressure, gestational diabetes, and postpartum depression. Also, being exposed to anything before birth has been linked to a higher chance of giving birth too early, having a low birth weight, having breathing issues, having problems with the immune system, and having cognitive impairments in new-borns. There are, however, discrepancies in the literature; other studies show that there is no association in some populations, which shows that additional research is needed.

The results of this study are in line with what other studies have shown on the links between air pollution and poor health outcomes for mothers and new-borns Gaskins *et al*.^33^ , Xu *et al*.^44^ observed that being around Particulate matter smaller than 2.5 microns and Nitrogen Dioxide increased the risk of miscarriage. However, a European study indicated that lifestyle and genetic factors had a bigger impact. Similarly, Yuan *et al*.^36^ and Sun *et al.*^35^ found that being around pollution made people more likely to have high blood pressure, although a Nordic study did not detect this relationship, perhaps because pollution control strategies were different. The link between prenatal exposure to air pollution and immune dysfunction in new-borns, found by Pedersen *et al.*^46^ and Martins Costa Gomes *et al.*^47^ supports the idea that environmental pollutants have a big impact on developmental health. However, studies that don't agree with this suggest that some adaptive immune mechanisms may lessen the long-term effects. These differences show how important it is to think about differences in genetics, socioeconomic status, and location when looking at findings.

This review's main result was that Particulate matter smaller than 2.5 microns and other types of air pollution can make it more likely for a woman to lose her baby. This link is in line with what Gaskins *et al*. and Xu *et al*., found: that being around more particulate matter and carbon monoxide made it more likely that a woman would lose her pregnancy early.^33^^,^^44^ Oxidative stress, which is a recognized effect of being around pollution, might be the cause of this. Oxidative stress can mess with hormonal signals and the function of the placenta, which might cause the fetus to die. For example, Particulate matter smaller than 2.5 microns exposures has been found to cause inflammation, which might affect blood flow to the uterus and placenta, which is important for keeping a pregnancy going. Also, research like Gaskins *et al.*^17^ backs up the idea that greater levels of Nitrogen Dioxide and Particulate matter smaller than 2.5 microns exposure led to higher rates of pregnancy loss, even in pregnancies that were helped by assisted reproductive technologies_._^43^ This demonstrates that pollution-related hormonal changes may impair both spontaneous and aided pregnancies. This shows how sensitive the reproductive system is to stresses in the environment.

Researchers have shown a strong association between exposure to pollutants such Particulate matter smaller than 2.5 microns, Nitrogen Dioxide, and black carbon and hypertensive diseases during pregnancy, such as preeclampsia. It is found that when a mother is exposed to these pollutants, her chance of having new high blood pressure problems during pregnancy goes up.^35^^,^^36^ Systemic inflammation and oxidative stress are probably what link air pollution with preeclampsia. Researchers have shown that pollutants including Particulate matter smaller than 2.5 microns and black carbon can cause the release of pro-inflammatory cytokines, which are thought to have a role in the development of preeclampsia. Pollutants like Particulate matter smaller than 2.5 microns can cause the body to produce interleukins (IL-6, IL-1β), which can contribute to endothelial dysfunction, a common sign of preeclampsia.^11^ In this case, studies evidence that being exposed to something during important times in pregnancy, like the first trimester, may have the worst effects on how well a woman's blood pressure is controlled.^35^^,^^36^ This result shows how important it is to think about when people are exposed in future research. 

This review also found a strong link between exposure to air pollution and preterm delivery and low birth weight, especially with pollutants such Particulate matter smaller than 2.5 microns, Particulate Matter ≤10 micrometers, and Nitrogen Dioxide. Researcher said that being around more Particulate matter smaller than 2.5 microns and Sulfur Dioxide during pregnancy greatly boosted the chance of having a baby too early or with a low birth weight.^34^^,^^41^ One possible explanation for these links is that pollution might create inflammation and oxidative damage that makes the placenta less effective. For instance, when a mother is exposed to pollutants, it can interfere with the development of the placenta by affecting blood flow, food transfer, and oxygen exchange. This can lead to fetal hypoxia, which is a known risk factor for low birth weight and premature birth.^12^ Also, research has indicated that air pollution can disrupt the DNA methylation of the placenta itself, which could have an effect on how the fetus grows. Ghazi *et al*.^50^ discovered that being exposed to Particulate matter smaller than 2.5 microns and Nitrogen Dioxide before birth changed the DNA methylation in the placenta, which can affect gene expression that is important for fetal development. This change in molecules could explain why some pollutants are linked to low birth weight and preterm birth: they interfere with normal fetal development at the genetic level.

Studies like those by Niedzwiecki *et al.*^37^ and Duan *et al*.^38^ are discovering more and more evidence that being around air pollution can lead to postpartum depression. According to this research, being around Particulate matter smaller than 2.5 microns through gestation & the first few months after giving birth raises the risk of depression. This might work in a way similar to how air pollution affects high blood pressure by changing the chemistry of the brain and how it controls mood. This research shows that environmental pollution is a foremost issue that has to be addressed since it impacts both the physical and mental health of mothers. Women who breathe in a lot of polluted air may be more likely to develop mental health problems, which might have long-term effects on the health of both the mother and the baby.

The effect of prenatal air pollution exposure on neonatal health, particularly respiratory and immune function, has been well-documented. Pedersen *et al*.^46^ and Martins Costa Gomes *et al.*^47^ demonstrated that exposure to pollutants like Nitrogen Dioxide & Particulate matter smaller than 2.5 microns through gestation alters immune cell profiles in neonates, making them more susceptible to respiratory diseases, allergies, and asthma. The immune system of a new-born is still developing, and prenatal exposure to pollutants may alter its development, leading to long-term health consequences. This finding is in line with existing research that links antenatal vulnerability to environmental pollution with immune dysfunction, highlighting the need for early interventions to protect infants from these environmental stressors. Also, research articles have revealed that environmental pollution might make new-borns’ lungs work worse, which makes them more likely to have wheezing and other breathing problems. Researchers showed that being exposed to Particulate Matter ≤10 micrometers and Nitrogen Dioxide before birth greatly impaired lung function and raised the incidence of wheeze in new-borns.^45^ This study shows how important it is to lower prenatal exposure to air pollution since the respiratory system is very sensitive to pollutants in the environment when a person is still developing. 

Strength and limitations. One of the best things about this systematic review is that it looks at 26 different studies in depth, giving it a worldwide perspective and a large sample size of 528 802 people. Adding different research designs makes the results more generalizable, and molecular insights from studies help us understand how biological processes work. However, limitations include methodological variability across studies, potential exposure misclassification, and residual confounding from unaccounted factors. Additionally, the heterogeneity of pollutants examined complicates conclusions, and the geographical and temporal variability across studies may limit the applicability of findings to current conditions. 

Conclusion. This systematic investigation corroborates the significant correlation between ambient air pollution and detrimental maternal and new-born outcomes, such as miscarriage, hypertensive disorders, preterm birth, and low birth weight. These findings provide essential insights for enhancing nursing care, facilitating early risk detection, maternal education, and the promotion of cleaner surroundings. Public health policy must mitigate exposure, while next research should concentrate on longitudinal cohort studies and targeted interventions to enhance evidence-based mother and child health nursing practices.
